# Role of Tumor-Associated Myeloid Cells in Breast Cancer

**DOI:** 10.3390/cells9081785

**Published:** 2020-07-27

**Authors:** Yoon Jin Cha, Ja Seung Koo

**Affiliations:** Department of Pathology, Yonsei University College of Medicine, Seoul 03722, Korea; yooncha@yuhs.ac

**Keywords:** breast cancer, tumor-associated myeloid cells, tumor-associated macrophage, myeloid-derived suppressor cells

## Abstract

Stromal immune cells constitute the tumor microenvironment. These immune cell subsets include myeloid cells, the so-called tumor-associated myeloid cells (TAMCs), which are of two types: tumor-associated macrophages (TAMs) and myeloid-derived suppressor cells (MDSCs). Breast tumors, particularly those in human epidermal growth factor receptor 2 (HER-2)-positive breast cancer and triple-negative breast cancer, are solid tumors containing immune cell stroma. TAMCs drive breast cancer progression via immune mediated, nonimmune-mediated, and metabolic interactions, thus serving as a potential therapeutic target for breast cancer. TAMC-associated breast cancer treatment approaches potentially involve the inhibition of TAM recruitment, modulation of TAM polarization/differentiation, reduction of TAM products, elimination of MDSCs, and reduction of MDSC products. Furthermore, TAMCs can enhance or restore immune responses during cancer immunotherapy. This review describes the role of TAMs and MDSCs in breast cancer and elucidates the clinical implications of TAMs and MDSCs as potential targets for breast cancer treatment.

## 1. Introduction

Breast cancer is one of the most common malignant tumors among women and a major cause of mortality among women worldwide [1]. Although the overall survival of breast cancer patients has improved owing to advancements in early detection and treatment methods, a subset of breast cancer, especially triple-negative breast cancer (TNBC), has revealed limited improvement in survival rates owing to the lack of effective treatment methods, except for surgery [2]. The tumor microenvironment (TME) contains potential therapeutic targets in such patients. The TME contains non-transformed host cellular components of the tumor mass residing within the tumor region, including immune system elements (including macrophages and lymphocytes), blood vessels, fibroblasts, myofibroblasts, mesenchymal stem cells, adipocytes, and extracellular matrix (ECM) components [3]. Among these, tumor-associated myeloid cells (TAMCs) are a subset of immune cells and are classified into tumor-associated macrophages (TAMs), myeloid-derived suppressor cells (MDSCs), tumor-associated neutrophils (TANs), Tie2-expressing monocytes (TEMs), and tumor-associated dendritic cells (TADCs). Among these, TAM and MDSC are the most abundant tumor-infiltrating immune cells. Human epidermal growth factor receptor 2 (HER-2)-positive breast cancer and TNBC commonly contain immune cells in their stroma. TAMs are the major TME component in breast cancer and potentially account for >50% of the TME [4]. The TME in breast cancer plays an important role in tumor development, progression, and metastasis [5,6], and TAMCs are further involved in physiological phenomena in breast tumors. Since TME elements are involved in various steps of tumorigenesis, the TME appears to be an attractive therapeutic target. This review discusses the various roles of TAMCs including TAMs and MDSCs in breast cancer and their clinical implications as therapeutic targets.

## 2. Definition and Classification of Tumor-Associated Myeloid Cells (TAMCs)

**Figure 1:**
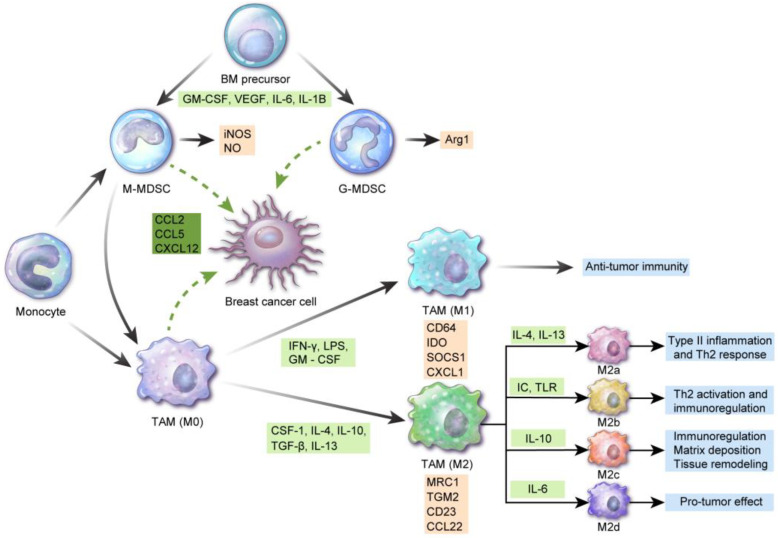
Differentiation and characteristics of tumor-associated myeloid cells: interferon (IFN)-γ, lipopolysaccharide, and granulocyte-macrophage colony-stimulating factor (GM-CSF) induce M1 tumor-associated macrophage (TAM) polarization from monocytes, which are involved in antitumor immunity. CSF-1, interleukin (IL)-4, IL-10, transforming growth factor (TGF)-β, and IL-13 contribute to M2 TAM polarization. M2 macrophages are further differentiated into M2a by IL-4 and IL-13 and are involved in type II inflammation and the Th2 response. Differentiation of M2 macrophages into M2b leads to Th2 activation and immunoregulation via immune complex and toll-like receptor ligand. M2c and M2d differentiation by IL-10 and IL-6 is involved in immunoregulation, matrix deposition and tissue remodeling, and induction and growth of tumor cell masses, respectively. Surface antigens of M1 macrophages include CD64, indoleamine 2,3-dioxygenase (IDO), suppressor of cytokine signaling 1 (SOCS1), and chemokine (C-X-C motif) ligand 1 (CXCL1). Mannose receptor C-type 1 (MRC1), transglutaminase 2 (TGM2), CD23, and C-C Chemokine Ligand 2 (CCL2) are considered M2 macrophage markers. Myeloid-derived suppressor cells (MDSCs) originate from bone marrow precursor cells in the presence of GM-CSF, vascular endothelial growth factor (VEGF), IL-6, and IL-1B and are divided into CD11b^+CD14^+HLA-DR^−/low^ CD15^−^ monocytic MDSCs and CD11b^+^CD14^–^HLA-DR^low/−^ CD15^+^ granulocytic MDSCs, the former secreting inducible nitric oxide synthase (iNOS) and NO and the latter releasing reactive oxygen species (ROS) and Arg1. Among these, monocytic MDSCs can differentiate to TAMs. In breast cancer, CCL2, CCL5, and CXCL12 are involved in TAM and/or MDSC recruitment.

TAMCs include TAMs and MDSCs, both of which can differentiate into different cellular subsets based on their microenvironment. Each of these cellular components also has unique characteristics and plays different roles (Figure 1).

### 2.1. TAMs

Macrophages are classified into recruited macrophages from the bone marrow and tissue-resident macrophages from the primitive yolk sac precursor, based on their origin [7,8]. In breast cancer, proliferation of tissue-resident macrophages determines the TAM pool [9]. TAMs are of two types, designated M1 (classically activated or proinflammatory “killer M1”) and M2 (alternatively activated, anti-inflammatory “builder M2”) [10]. The M1 type is associated with the Th1 response involved in eliminating intracellular pathogens and antitumor immunity [11,12]. The M2 type is further classified into types M2a, M2b, M2c, and M2d [13]. The M2a type is associated with type II inflammation, i.e., the Th2 response along with IL-4 and IL-13 release, and with the response against parasitic infections [14]. The M2b type contributes to Th2 activation and immunoregulation via the immune complex and toll-like receptor ligands [15]. The M2c type induces immunoregulation, matrix deposition, and tissue remodeling via interleukin (IL)-10 [16]. The M2d type is activated by IL-6 and enhances the induction and growth of tumor cell masses through angiogenesis [17]. Another study has suggested macrophage nomenclature based on the activating factors including M(IL-4), M(Ig), M(IL-10), M(GC), M(IFN-), and M(LPS) instead of M1/M2 terms [18]. M1 macrophages express CD64, indoleamine 2,3-dioxygenase (IDO), suppressor of cytokine signaling 1 (SOCS1), and chemokine (C-X-C motif) ligand 1 (CXCL1), and M2 macrophages express mannose receptor C-type 1 (MRC1), transglutaminase 2 (TGM2), CD23, and CCL22 [19]. M1 and M2 macrophages do not have fixed phenotypes and may vary in accordance with different external stimuli. Various hormones, cytokines, and apoptotic cells affect macrophage polarization [20,21]. Generally, interferon (IFN)-γ, lipopolysaccharide (LPS), and GM-CSF are involved in M1 polarization of monocytes, while CSF-1, IL-4, IL-10, transforming growth factor (TGF)-β, and IL-13 are involved in M2 polarization. Furthermore, M1 polarization is induced by two signals from the TME including IFN-γ and toll-like receptor ligand [22,23]. M1 and M2 macrophages can undergo mutual transformation [24]. “M1-like” macrophages have cytotoxic function and antitumor activity, whereas “M2-like” macrophages are associated with tumor progression, repair, and immunosuppression [18]. Gene expression profiling data have reported the M2-like nature of TAMs in breast cancer [25,26,27]. Breast cancer cells secrete molecules inducing the M2-like phenotype in TAMs [28], especially in basal-like breast cancer [29]. Regulation through miRNA is another mechanism for TAM polarization, wherein miR-146a promotes the M2 phenotype [29]. Furthermore, M2-like polarization has been observed in brain metastasis in breast cancer [30]. TAMs in breast cancer comprise several subgroups and display intra-tumoral heterogeneity. TAMs with the M1-like phenotype are CD206^-^Dextran^-^MHC-II^high,^ migratory TAMs located in the perivascular area and display pro-metastatic features. However, M2-like TAMs are CD206^+^Dextran^+^MHC-II^low^ sessile TAMs at invasive borders and hypoxic areas and display pro-angiogenetic features [31]. M1 macrophages in breast cancer are associated with increased cancer cell apoptosis, decreased metastasis, and cancer invasion [32,33].

### 2.2. MDSCs

MDSCs are composed of heterogenous immature myeloid cells and suppress the immune response. MDSCs are classified into monocytic-MDSCs (CD11b^+^CD14^+^HLA-DR^−/low^ CD15^−^) and granulocytic-MDSCs (CD11b^+^CD14^−^HLA-DR^low/−^ CD15^+^) [34]. Heterogeneity is caused by tumor-derived soluble factors, which are involved in myelopoiesis and MDSC recruitment [35] and affect MDSC function under specific microenvironments [36]. Composition of the MDSC subset is changed according to the type of tumor [37]. Immunosuppressive mechanisms in tumor cells via MDSCs are as follows: monocytic-MDSCs express inducible nitric oxide synthase (iNOS) and generate nitric oxide (NO), while granulocytic-MDSCs produce reactive oxygen species (ROS) and arginase 1 [38]. Subsequently, amino acid 1-arginine depletion and T cell receptor (TCR)-chain are suppressed, resulting in cell cycle arrest [38]. ROS and NO production induce TCR peroxynitration and T cell apoptosis [39]. MDSCs secrete immunosuppressive cytokines including IL-10 and TGF-β [40,41], inducing regulatory T-cells [42] and influencing natural killer (NK) cell function [43]. Moreover, MDSC activation induces the expression of PD-L1 and immune suppression [44].

MDSCs from breast cancer patients are functionally and phenotypically similar to bone marrow-derived MDSCs, suggesting that breast cancer MDSCs originate from bone marrow precursors [45]. Cytokines and chemokines promote MDSC accumulation at tumor sites in breast cancer. These cytokines include IL-6 [46], IL-1β [47], G-CSF [48], M-CSF [49], GM-CSF [50], macrophage Migration Inhibitory Factor (MIF) [51], and TGF-1β [52], and the reported chemokines are CXCL5 [53], CCL1 [47], CCL2 [54], and CCL5 [55].

### 2.3. Association Between TAMs and MDSCs in the TME

TAMs and MDSCs are different cell types but are not clearly distinguished and share several common characteristics. Local macrophages within tissues generally originate from monocytes of embryonic tissue or bone marrow. Two types of monocytes are present in the blood: patrolling monocytes and inflammatory monocytes. In the TME, cytokines, chemokines, and metabolites from tumor cells can influence normal myelopoiesis [56] and can increase the differentiation of monocytic MDSCs into granulocytic MDSCs. Monocytic MDSCs and inflammatory monocytes migrate to the tumor area via the CCL2/CCR2 and CSF pathways and are differentiated into TAMs through various factors secreted by tumor cells. Thus, MDSCs are involved in TAM differentiation. In breast cancer, Ly6ChiCX3CR1^low^ monocytes [27] and angiopoietin receptor Tie-2-positive monocytes [57,58] are potential TAM precursors.

## 3. Role of TAMs in Breast Cancer

**Figure 2:**
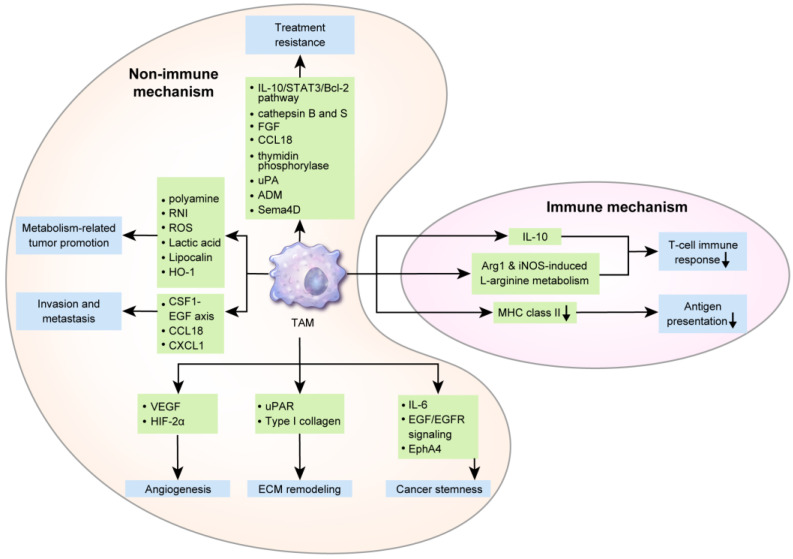
The role of tumor-associated macrophages (TAMs) in breast cancer: One of the immunogenic mechanisms underlying the secretion of IL-10, Arg1, and iNOS-related L-arginine by TAMs in breast cancer, which suppress the T-cell response and antigen presentation by decreasing major histocompatibility complex (MHC) class II levels. Non-immunogenic mechanisms include angiogenesis via the secretion of VEGF and hypoxia-inducible factor (HIF)-2α; extracellular matrix remodeling via releasing urokinase receptor (uPAR) and type I collagen; and evoking cancer stemness through IL-6, epidermal growth factor (EGF)/EGF receptor(EGFR) signaling, and EphA4. TAM contributes to invasion and metastasis via the CSF1-EGF axis, CCL18, and CXCL1. Polyamine, reactive nitrogen intermediates (RNI), ROS, lactic acid, lipocalin (LCN), and heme oxygenase-1 (HO-1), which are TAM metabolites, also promote breast cancer progression. Finally, treatment resistance mechanisms via TAMs are supported by the IL-10/STAT3/Bcl-2 pathway, cathepsin B and S, fibroblast growth factor, CCL18, thymidine phosphorylase, urokinase-type plasminogen activator (uPA), adrenomedullin (ADM), and Sema4D.

TAMs affect breast cancer cells via various mechanisms influencing breast cancer initiation, progression, metastasis, and responses to therapeutic agents. Hence, TAMs can also influence breast cancer prognosis. In a breast cancer epidemiological study, a larger number of TAMs was correlated with a poor clinical prognosis [59]. Furthermore, a meta-analysis reported that a higher TAM density was significantly associated with a worse relapse-free survival (RFS) and overall survival (OS) [60]. Several mechanisms underlying the effects if TAMs on a poor breast cancer prognosis are described below (Figure 2).

### 3.1. Immune Mechanism of TAMs in Breast Cancer Progression

Suppression of antitumor T-cell responses by anti-inflammatory cytokines secreted by TAMs is one of the mechanisms of tumor immune evasion. In previously reported animal models of breast cancer, TAM-derived IL-10 suppressed CD8^+^T-cell activation [61,62] and IL-12 secretion from dendritic cells inhibited CD8^+^T-cell responses by dendritic cells [61]. TAM-secreted Arg1 catabolized L-arginine, and the reduced L-arginine levels repressed effector T-cells [63]. In a mouse model of early-stage breast cancer, Arg1 was upregulated in TAMs [64]. iNOS is upregulated in TAMs and is involved in L-arginine metabolism, and Arg1 and iNOS from TAM inhibited T-cell responses in murine mammary tumors [65]. Furthermore, decreased tumoricidal TAM function contributes to tumor immune evasion. In macrophages derived from mouse breast cancer tissue, IL-12 and iNOS were downregulated and are important for the distribution of cancer cells [66,67]. Macrophages associated with murine mammary cancer line 4T1 and human breast cancer line MDA-MB-231 display MHC class II downregulation, thus reducing the immune-related antigen presentation [68]. TMAs potentially interact with other immune cells within the TME. Activated neutrophils secrete IL-8 and TNF-α, which can recruit macrophages, and myeloperoxidase (MPO) from neutrophils binds to macrophages mannose receptor (MMR) [69], which is upregulated in M2-like macrophages [70]. In human breast cancer tissue, MPO-positive neutrophils were observed in up to 16% of cases, and were associated with an enhanced prognosis [71], probably owing to the activation of M2-like macrophages induced by MPO-positive neutrophils.

### 3.2. Nonimmune Mechanism of TAMs in Breast Cancer Progression and Metastasis

TAMs initially affect breast cancer progression first through angiogenesis. Hypoxia upregulates VEGF and HIF-2α in TAMs in human breast cancer tissue [72,73]. In a previous study, breast cancer spheroids from the human T47D breast tumor cell line, including macrophages, were transplanted into mice, which resulted in angiogenesis through VEGF overexpression [74]. In breast cancer in humans, TAM infiltration increases with an increase in angiogenesis [75,76]. In a gene expression profiling study, TAMs obtained from late-stage breast cancer secreted 2-fold levels of angiogenesis mediators compared to control cells [25]. Furthermore, TAMs affect breast cancer progression by remodeling the ECM. Human breast invasive ductal carcinoma (IDC) and ductal carcinoma in situ (DCIS) display higher urokinase receptor (uPAR) expression levels in TAMs in the peritumoral region. Interaction with urokinase-type plasminogen activator (uPA) elicits plasminogen-dependent proteolysis, resulting in matrix remodeling and cancer cell migration [77]. In mice, macrophage deficiency during breast cancer pathogenesis reduced type I collagen production and additional macrophage administration restored type I collagen production [78]. In addition, cancer stem cells (CSCs) are activated by various cytokines from TAMs [79], affecting breast cancer prognosis. IL-6 from TAMs promote an inflammatory environment associated with the prolongation of CSC-like features of tumor cells in the premalignant stage, primary tumors, and metastatic stage [79]. Co-culture of ER-positive breast cancer cell lines and M2 macrophages enhanced tumor sphere formation [80], and in mouse models, TAM was associated with the promotion of SOX2, a CSC regulatory factor in EGF/EGFR signaling [81]. A proteomics study revealed that TAMs interact with breast CSCs in mediating EphA4 responses, thus enhancing tumorigenesis and facilitating CSC maintenance [82].

Furthermore, TAMs are involved in breast cancer metastasis. Human breast cancer tissue with lymph node metastasis has been reported to have an increased number of CD68^+^ TAMs [83] and VEGF-C^+^ TAMs [84]. In human TNBC tissue, the number of TAMs was correlated with the risk of distant metastasis [85]. TAM is involved in intravasation, which is an important step in breast cancer metastasis. In animal models, perivascular macrophages contribute to breast cancer intravasation [86], which was assisted through positive feedback interactions between EGF from TAM and CSF1 from breast cancer cells [86,87]. Through CSF1-EGF interactions, invadopodia and podosome were formed in breast cancer cells and TAMs, respectively, thus promoting ECM breakdown and intravasation [86,87]. Moreover, breast cancer cell intravasation was induced through integrin clustering via CCL18 from TAMs in breast cancer cell lines and human breast cancer tissue [88]. Furthermore, TAMs are involved in seeding and site-specific cancer cell metastasis. In the mouse model, VEGFR1^+^CCR2^+^CX3CR1^+^Tie2^–^CXCR4^−^ macrophages were associated with tumor cell seeding [26,89]. Tumor seeding occurred through an adherent scaffold, which was formed by breast cancer cell-derived lysyl oxidase (LOX)-mediated linkage of macrophages and collagen type IV in the bone marrow and lungs in previously described mouse xenograft models [90,91]. In mouse models of breast cancer, the recruitment of CD11b-positive macrophages by CCL2 was reported to develop lung metastasis [92]. CXCL1 secreted from TAMs enhanced metastasis via nuclear factor (NF)-κB/SOX4 activation in mouse and human breast cancer cell lines [93].

### 3.3. Metabolic Interactions of TAMs with Cancer Cells

Tumor cells display altered metabolism, called the Warburg effect, wherein glycolysis rather than oxidative phosphorylation is used for energy production [94]. Furthermore, TAMs have altered metabolism. Arg1 is upregulated in M2 macrophages, which converts L-arginine into L-ornithine and polyamine. NOS is upregulated in M1 macrophages, which converts L-arginine to NO and L-citrulline [95]. Previous studies using breast tumor cells (ZR-75-1) reported that ARG1-mediated polyamine production in TAMs increased tumor cell proliferation [96]. Under hypoxic conditions, HIF-1α upregulation in TAMs is reportedly associated with glycolysis [97,98] and activated HIF-1α induces genetic alterations and tumorigenesis by producing reactive nitrogen intermediates and ROS [99]. Lactic acid produced during glycolysis in TAMs induce TAM polarization to tumor-promoting cells [100] or immunosuppressive and pro-angiogenic phenotypes contributing to tumor progression [101]. In an MMTV-PyMT breast cancer model, TAM were polarized into M2 phenotype by Th2 cell-derived IL-4 [102]. As IL-4 promotes oxidative phosphorylation in macrophages [103], TAM metabolism in breast cancer may proceed through oxidative phosphorylation rather than glycolysis. TAM generally displays a phenotype similar to that of M2 macrophages; however, polarization could depend on the type of tumor or on tumor progression. Hence, metabolic features may accordingly differ.

Furthermore, TAMs may display alterations in lipid metabolism. TAMs expressing epidermal fatty acid-binding proteins (E-FABP) reportedly suppress tumor growth by increasing the IFN-β reaction induced by an increase in lipid droplet formation in mouse models of breast cancer [104]. Lastly, iron metabolism in TAMs potentially influences tumor cells. The iron exporter, ferroportin, and H-ferritin are generally upregulated in M1 macrophages. However, M2 macrophages display the opposite phenotype of high ferroportin and low H-ferritin levels [105]. TAM displayed increased secretion of lipocalin (LCN), an iron-releasing protein, which induces the proliferation in the breast cancer cell line MCF-7 [106]. In a previously used mouse breast cancer model, TAM-releasing heme oxygenase-1 (HO-1), an iron-releasing enzyme, enhanced breast cancer growth [107].

### 3.4. Induction of Treatment Resistance by TAMs

In the TME, TAMs and cancer cells differently respond to breast cancer treatment [108]. TAM polarization affects the degree of influence of TAMs on treatment responses to chemotherapy [108,109]. Treatment resistance was associated with high M2 macrophage numbers, whereas treatment responses to docetaxel were associated with the depletion of M2 TAMs and expansion of M1 TAMs in 4T1-Neu mammary tumor-bearing mice [110]. TAM-derived IL-10 display increased expression of bcl-2 and STAT3 genes, and the subsequent IL-10/STAT3/Bcl-2 signaling pathway induced TAM-mediated treatment resistance on co-culturing human breast cancer cell lines (T47D, BT549) and TAMs (THP-1) [111]. TAMs are associated with tamoxifen resistance in postmenopausal breast cancer patients [112]. Secretion of chemoprotective molecules including cathepsin B and S has been suggested as a mechanism underlying TAM-mediated treatment resistance in a PyMT mouse model of breast cancer [113]. Furthermore, TAM repressed the recruitment of CD8^+^ cytotoxic T-cells, resulting in drug resistance in the MMTV-PyMT breast cancer model [102]. Various molecules including basic fibroblast growth factor, chemokine CCL18 [114], thymidine phosphorylase [115], urokinase-type plasminogen activator (uPA) [116], adrenomedullin (ADM) [117], and semaphorin 4D (Sema4D) [118] reportedly enhance angiogenesis and inhibit immune responses, thus resisting the antiangiogenesis agent. Hence, inhibition of TAM-derived angiogenesis-inducing factors potentially improve the efficacy of chemotherapy [119,120]. Recent study, regarding immune checkpoint blockad with chemotherapy in TNBC patients, reactive oxygen species (ROS) and oxidative stress induced by taxane in macrophages render them immunosuppressive and expressing PD-L1 [121].

## 4. Role of MDSCs in Breast Cancer

**Figure 3:**
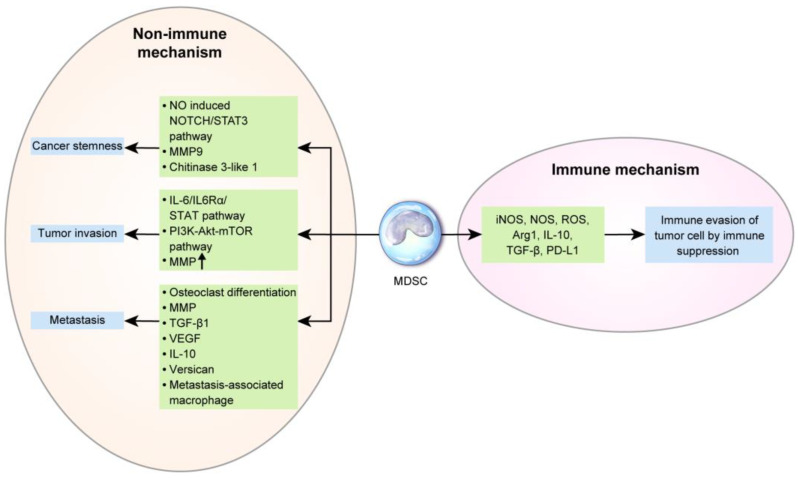
The role of myeloid-derived suppressor cells (MDSCs) in breast cancer: Common immunogenic pathways of MDSCs in breast cancer progression are the induction of immunosuppression by iNOS, NOS, ROS, Arg1, IL-10, TGF-β, and PD-L1, thus facilitating immune evasion of tumor cells. Non-immunogenic mechanisms include the enhancement of cancer stemness by the nitric oxide (NO)-induced Notch/ signal transducer and activator of transcription 3 (STAT3) pathway, matrix metallopeptidase (MMP) 9, and chitinase 3-like 1 and the promotion of tumor invasiveness by the IL-6/IL6Rα/STAT pathway, phosphoinositide 3-kinase (PI3K)-Akt-mammalian target of rapamycin (mTOR) pathway, and MMP upregulation. During metastasis, MDSCs differentiate into osteoclasts, which increases osteolytic bone metastasis and promotes MMP, TGF-β1, VEGF, IL-10, and versican secretion, and into metastasis-associated macrophages.

In breast cancer, based on the tumor type, MDSCs are recruited to the tumor sites by various chemokines [122] including CCL2 [123], CXCL5 [124], and CXCL12 (SDF-1) [125]. MDSC levels are increased in breast cancer patients and are associated with the clinical stage and metastatic disease burden [126,127]. In particular, the number of monocytic MDSCs is associated with the metastasis status of breast cancer [128]. Furthermore, MDSC levels are associated with a shorter OS in metastatic breast cancer [129]. MDSC levels can change upon treatment. A previous study reported that granulocytic MDSCs are significantly decreased after chemotherapy [126]. Baseline MDSC levels are significantly lower in chemo-responsive patients [130]. The mechanisms underlying the prognosis and treatment responses of MDSCs in breast cancer are described below (Figure 3).

### 4.1. Immune Mechanism of MDSCs in Breast Cancer Progression and Metastasis

The basic function of MDSC is immunosuppression in tumors and in the normal state. The mechanism underlying MDSC-mediated immunosuppression is described above. Previous studies have encountered challenges in isolating MDSCs from tumors [131]; however, new methods including magnetic-activated cell sorting (Miltenyi biotec, Bergisch Gladbach, Germany) have facilitated the separation of MDSCs from tumor tissue and further analyses. The difference between MDSCs of peripheral lymphoid organs and tumors are as follows: in peripheral lymphoid organs, cell-to-cell contact is important in MDSC-mediated T-cell inhibition [132]. However, in tumors, monocytic MDSCs use NO, Arg1, and immunosuppressive cytokines, which have a long half-life and no requirement for close contact, for non-pecific and higher immunosuppression [133,134,135]. In tumors, MDSCs have a different mechanism of action based on the tumor type, resulting in different ratios of monocytic and granulocytic MDSCs. Most cancers, including breast cancer, display predominant granulocytic MDSCs in peripheral blood [136]; however, prostate cancer has a higher proportion of monocytic MDSCs than of granulocytic MDSCs [37]. Granulocytic MDSCs in breast cancer are activated by IL-17 from tumor-infiltrating γδ T-cells and inhibit CD8^+^T-cells, thus enhancing lymph node and lung metastasis [137]. However, most tumor tissues have markedly higher proportions of monocytic MDSCs [138,139]. In breast cancer, MDSCs affect tumor cells by interacting with NK cells. In a previously described murine model of breast cancer, interactions between MDSC (CD11b+/Ly6Cmed/Ly6G+) and NK cells (CD3-/NK1.1+) promoted metastasis by significantly reducing the cytotoxicity of NK cells against tumor cells [140].

### 4.2. Nonimmune Mechanism of MDSCs in Breast Cancer Progression and Metastasis

Interaction between breast cancer cells and MDSCs is important in breast cancer progression. Cancer cell-derived IL-6 activates the STAT3 pathway in MDSCs, subsequently upregulating indoleamine 2,3-dioxygenase in co-cultures of human CD33(+) myeloid progenitors with MDA-MB-231 breast cancer cells [141]. G-CSF and GM-CSF from cancer cells activate the STAT3 and STAT5 pathways, which repress interferon regulatory factor-8, as reported in a previous mouse model of breast cancer [142]. TGF-1β from breast cancer cells activates miRNA-494 in MDSCs, which inhibit phosphatase and tensin homolog (PTEN) and activate the AKT pathway, as reported in a previous mouse model of breast cancer treated with a human breast cancer cell line [52]. Furthermore, MDSCs in breast cancer are associated with CSC. The number of tumor-infiltrating CD33-positive MDSCs is associated with aldehyde dehydrogenase (ALDH)-positive CSCs in breast cancer patients. NO released from MDSCs activates Notch and signal transducer and activator of transcription 3 (STAT3) in breast cancer cells, enhancing breast cancer stemness in co-cultures of human breast cancer cells and MDSCs [143]. Breast cancer cell-derived IL-6 promotes IL-6 and IL6Rα expression in MDSCs, and IL-6 trans-signaling in cancer cells activates STAT phosphorylation, thereby promoting breast cancer invasion and metastasis in a mouse model established through a xenograft of human breast cancer cells [46,144]. The AKT pathway is activated in MDSCs activated by breast cancer cells, and MMPs are upregulated, which promote cancer cell invasion and metastasis, as reported in a mouse model treated with a human breast cancer cell line [52]. CCL3 secreted by breast cancer cells recruits MDSCs, which activates the PI3K-AKT-mTOR pathway in breast cancer cells, thereby inducing the epithelial-mesenchymal transition (EMT) and tumor cell migration and invasion in co-cultures of human breast cancer cell lines and MDSCs [145].

Furthermore, MDSCs serve as osteoclast progenitors in breast cancer and enhance cancer-associated osteolysis. MDSCs differentiate into osteoclasts through NO signaling and cancer cells, thereby promoting osteolysis during bone metastasis in breast cancer, as reported in a murine model of breast cancer [49,146,147]. In breast cancer, the SDF-1/CXCR4 and CXCL5/CXCR2 axes recruit Gr-1^+^CD11b^+^ myeloid cells, activate MMP, and upregulate TGF-β1, which contributes to metastasis in mouse models injected with a breast cancer cell line (4T1) [124]. MDSCs are involved in metastasis-associated macrophages in mouse breast cancer model. Monocytic MDSCs (Gr1-positive inflammatory monocytes) are recruited by the CCL2-CCR2 axis in pulmonary metastasis and differentiated into metastasis-associated macrophages, which facilitate extravasation, seeding, and tumor cell growth in a PyMT mouse model of breast cancer [148]. In a previously reported mouse model injected with breast cancer cell line (4T1), among the recruited bone marrow-derived CD11b+Gr1+ myeloid progenitor cells in premetastatic lung, CD11b^+^Ly6C^high^ monocytic MDSCs secreted versican, an extracellular matrix proteoglycan, thus triggering the EMT, increases tumor cell proliferation, and accelerates metastasis [149]. MDSCs in breast cancer show increased secretion of TGF-β, VEGF, and IL-10, which induces EMT and metastasis [150]. TNBC is characterized by ΔNp63 upregulation, which activates CXCL2 and CCL22 and MDSC recruitment. MDSC enhances cancer stemness in TNBC via MMP9 and chitinase 3-like 1 secretion, which promotes metastasis, as reported in a in mouse model injected with the breast cancer cell line HCC1806 [151].

## 5. Targeting TAMCs for Breast Cancer Treatment

Breast cancer has immunogenic properties, similar to other solid tumors, which are elicited through TAMCs. Immunotherapy is a newly emerged treatment alternative in the field of promising antitumor therapy. Previous studies have attempted to assess the efficiency of immunotherapy in breast cancer: cancer vaccines, bispecific antibody (bsAb), immune checkpoint antagonist, and targeting lymphocyte activation gene-3 (LAG-3). Cancer vaccines use specific breast cancer antigens, including HER-2 [152] and MUC-1 [153], which activate tumor-specific T-cells. Among cancer vaccines, cell-based vaccines (Lapuleucel-T) target her-2/Neu positive breast cancer cells containing tumor antigens [154]. BsAb simultaneously reacts with two tumor antigens—CD3 and HER-2 [155]. Immune checkpoint antagonists include cytotoxic T-lymphocyte-associated protein 4 (CTLA-4) blockers (tremelimumab [156] and ipilimumab [157]) and PD-1/PD-L1 blockers (avelumab [158], atezolizumab [159], and pembrolizumab [160,161]), which have been previously studied in breast cancer. Lastly, LAG-3 is an MHC-II receptor and is expressed on T-cells, NK cells, and dendritic cells. LAG-3 suppresses T-cell activation and is involved in Treg immunosuppression [162]. IMP321 is a soluble form of LAG-3, which has been previously investigated in clinical trials on breast cancer patients [163].

The subsets of TAMCs (TAMs and MDSCs) are involved in various stages of breast cancer progression, including tumor development, progression, metastasis, and treatment responses (Table 1).

Thus, targeting of TAMs and MDSCs is effective in breast cancer in preclinical and/or clinical studies (Table 2).

### 5.1. TAMs as a Therapeutic Target

TAMs are a potentially effective therapeutic target, since they are involved in tumor progression, immune evasion, and treatment resistance in breast cancer [189]. TAMs have been assessed as a therapeutic target in preclinical and clinical studies, some of which have been proven effective.

#### 5.1.1. Inhibition of TAM Recruitment

Considering that high levels of TAMs in breast cancer are associated with a poor prognosis, inhibition of TAM recruitment may be considered a treatment alternative. Generally, TAM recruitment in the tumor area is regulated by macrophage chemoattractants secreted by cancer cells or the TME [109]. CCL2 is a representative chemoattractant that recruits TAMs via the CCL2-CCR2 axis [175]. CCL-2 blockade with anti-CCL2 antibody suppresses tumor growth and dissemination in breast cancer [190,191]. A preclinical study involving breast cancer patients assessed with carlumab (CNTO888), a monoclonal antibody against CCL-2, reported that carlumab was well-tolerated and displayed antitumor activity [166,167]. Agents targeting macrophage chemotactic factors, CCL5 and CXCL12, have displayed anticancer effects in estrogen receptor-positive breast cancer [192]. In TNBC, the anti-cathepsin D antibody decreases TGFβ levels and suppresses tumor growth by inhibiting TAM recruitment [168].

#### 5.1.2. TAM-Killing Drug

Chemical or synthetic drugs have been developed to directly eliminate TAMs [193]. RNA aptamers inhibit the murine or human IL-4 receptor-α (IL4Rα or CD124) and effectively deplete TAMs, thereby inhibiting tumor cell growth [194]. Trabectedin is a licensed and commercially available agent that selectively eliminates TAMs through caspase 8-dependent apoptosis via TRAIL receptors [195]. In the mouse tumor model, M2pep harboring a proapoptotic peptide displayed selective reduction of TAMs and displayed improved survival [170].

#### 5.1.3. Modulator of TAM Polarization and Differentiation

M2 TAMs are mostly involved in tumor progression, whereas M1 TAMs are involved in tumor suppression. Because of TAM plasticity, M1 or M2 TAMs can be switched through environmental stimulation [175]. Hence, conversion of M2 TAMs to pro-immunity antitumor M1 TAMs, which is regulated by the CSF1-CSF1R pathway, could be an effective therapeutic strategy [175]. The CSF1/CSF1R signaling pathway differentiates myeloid progenitor cells into mononuclear phagocytes, regulates TAM polarization, and enhances macrophage survival [196]. CSF1 or CSF1R overexpression in postmenopausal breast cancer is reportedly associated with a poor prognosis [197]. CSF1 loss in a breast cancer model displayed reduced tumorigenesis and inhibited tumor progression and metastasis [175]. Accordingly, RG7155, a monoclonal antibody inhibiting the CSF1 tyrosine kinase receptor, is a potential therapeutic agent for breast cancer [171]. Activation of toll-like receptor potentially induces the M1 TAM phenotype, as LPS and the toll-like receptor agonist promoted the polarization of M1 TAMs [198,199]. Zoledronic acid could change M2 TAMs into M1 TAMs and inhibited carcinogenesis in breast cancer [172]. Phenotypic conversion from M2 to M1 TAMs inhibited tumor growth and angiogenesis in breast cancer [28]. Liposomal nanoparticle-delivered guanosine monophosphate–adenosine monophosphate (GAMP) suppresses the growth of TNBC through reprograming from the pro-tumorigenic M2-like phenotype to the M1-like phenotype [173]. The C-terminal fragment of adhesion protein Fibulin7 suppresses the growth of a breast cancer cell line (MDN-MB-231) by reprogramming human monocytes to immunosuppressive TAMs [174]. Inhibition of M2 TAM polarization could also be a therapeutic strategy, and NF-κB, STAT3, STAT6, c-Myc, and interferon regulatory factor 4 are potential inhibitory factors [200]. Anti-CD40 monoclonal antibodies (mAbs) induce M1 TAM polarization [201]. Immunomodulation agents, thymosin-α and β-glucan, can also induce M1 TAM polarization [176]. *Hedyotis diffusa* and *Scutellaria barbata* herb couple (YDW11) inhibits M2 TAM polarization, thereby suppressing breast cancer cell migration [177].

#### 5.1.4. Reduction of TAM Products

The XIAOPI formula is an inhibitor of CXCL1, which is released from TAMs and inhibits the formation of a premetastatic niche in breast cancer [178] and breast cancer cell proliferation and metastasis [202]. ZnPPIX, a specific inhibitor of HO-1 and heat shock protein 32 (HSP32), effectively suppresses breast cancer cell growth [107].

### 5.2. MDSCs as a Therapeutic Target

MDSCs play an important role in various steps of breast cancer development and progression, thus rendering them a potential therapeutic target. Before targeting MDSCs for breast cancer treatment, one should consider several issues. Firstly, monocytic MDSCs are predominant in tumors rather than granulocytic MDSCs and rapidly differentiate into TAMs. Hence, specific MDSC-targeting agents would not be effective. Secondly, in the cancer setting, functional regulation of MDSCs would be different from that under normal physiological conditions. In a mouse tumor model, STAT3 inhibition induced MDSC depletion in the spleen but not in tumors [203]. Strategies targeting MDSCs are discussed below.

#### 5.2.1. Inhibition of MDSC Formation and Recruitment

To inhibit MDSC formation, cytokines and chemokines involved in MDSC differentiation should be inhibited. Curcumin, an IL-6 inhibitor, inhibits IL-6 secretion in breast cancer cells and reduces the number of MDSCs [179]. BMP4, a TGF-β growth factor family protein, reduced G-CSF in breast cancer cells [48]. R84 is a VEGF inhibitor and downregulates IL-1β, IL-6, and CXCL1 [47]. Sulforaphane and SB-265610 are respective inhibitors of MIF and CXCR2, and they can suppress MDSC formation and migration [51,53]. Silbinin suppresses CCR2 expression in MDSC, inhibits MDSC recruitment at tumor sites, and inhibits breast cancer growth [180]. NG-monomethyl-L-arginine acetate (L-NMMA) is an iNOS inhibitor, and it effectively inhibits MDSC-mediated osteolysis by inhibiting the differentiation of MDSCs into osteoclasts [49,147]. The anti-CCL5 antibody reportedly decreased MDSC activity and evoked T-cell proliferation, which could be an effective therapeutic strategy for TNBC [55]. HuMax-LI8, a monoclonal antibody neutralizing IL-8, reduces granulocytic MDSC recruitment and enhances immune-mediated tumor cell elimination by NK and antigen-specific T-cells in claudin-low type TNBC [181].

#### 5.2.2. Elimination of MDSCs

Activated T-cells with the anti-CD3/HER-2 bispecific antibody reportedly depleted MDSCs in breast cancer [182]. Attenuated bacterium *Listeria monocytogenes* (Listeria^at^) caused infections in MDSCs and reduced their number, thus drastically reducing the number of metastatic sites and tumor growth in breast cancer [183]. Reductions in the number of MDSCs could be achieved by herpes simplex virus 1 vector expressing 15-prostaglandin dehydrogenase (15-PGDH), through conversion of prostaglandin E2 into inactive 15-keto-metabolites by 15-PGDH, resulting in decreased ectopic primary and metastatic breast cancer [184]. Elimination of MDSCs is possible through the modulation of MDSC differentiation. All-trans retinoic acid and vitamin D enhanced MDSC differentiation [204]. Changes in MDSC phenotypes could repress their function by binding cyclic di-guanylate (c-di-GMP) and the stimulator of interferon genes (STING) on the MDSC surface in metastatic breast cancer [185]. Doxorubicin eliminates MDSCs through apoptosis via the ROS pathway in breast cancer [186]. A decreased number of MDSCs was also reported using phosphodiesterase 5 inhibitor [205] and celecoxib, a COX-2 inhibitor [187].

#### 5.2.3. Reduction of MDSC Products

Suppression of MDSC functional products is another therapeutic strategy. 1-methyl-L-tryptophan (1-MT) repressed IDO from MDSCs and resulted in repression of immune suppressive function of MDSCs against T-cell [188]. ROS is also a product of MDSC-mediated repression of CD8^+^T-cell responses. Thus, ROS inhibitors may suppress tumor progression. NOV-22, a glutathione disulfide mimetic, was effective in a clinical trial among breast cancer patients [130].

## 6. Conclusions

Breast cancer is one of the most important cancers among women and contains an immune cell stroma. Numerous HER-2-positive breast cancer and TNBC tissues contain immune cell stroma, and TNBC is a complex group composed of heterogenous subtypes with no specific target agent. TAMCs are the most popular compartment of immune cell stroma of breast cancer and is categorized into TAMs and MDSCs. Both these cell types contribute to breast cancer progression through immune and/or nonimmune mechanisms. One immune mechanism in both TAMs and MDSCs is immunosuppression, which facilitates immune evasion among cancer cells. Nonimmune mechanisms of TAMs include angiogenesis, extracellular matrix remodeling, metabolic support, induction of cancer stemness, and drug resistance. MDSCs promote breast cancer progression by increasing cell proliferation, cancer stemness, enhancing invasiveness by MMP and EMT, osteolytic metastasis via osteoclastic differentiation, and differentiation into metastasis-associated macrophages. Hence, targeting TAMs and MDSCs could be an effective therapeutic strategy, along with the immunotherapy, which has emerged as a popular treatment alternative. Thus far, agents targeting TAMs and MDSCs are antibodies, aptamers, and antagonists that suppress TAMC recruitment, deplete TAMCs, and modulate TAMC polarization and/or differentiation. Preclinical and/or clinical studies on breast cancer have reported the effectiveness of TAMC-targeting agents; however, certain points should be considered. First, TAMs and MDSCs have many cell subtypes from polarization and/or differentiation, having different functions and characteristics. Furthermore, it depends on the tumor subtype and degree of tumor progression. Hence, the types and distribution of TAMs and MDSCs in breast cancer should be investigated in accordance with the breast cancer subtype, tumor stage, primary or metastatic tumor tissue, and metastatic sites for precise target therapy. Second, adjuvant treatment modality including chemotherapy and radiotherapy could affect TAMCs [206,207,208,209,210], or TAMCs itself could elicit resistance to adjuvant therapies [211]. Furthermore, TAMC-targeting agents influence responses to chemotherapy and radiotherapy [212,213,214]; these secondary effects should be considered when adjuvant therapies and TAMC-targeting agents are concurrently applied. Lastly, TAMCs have variable physiological functions other than immunological functions, resulting in side effects of TAM-targeting agents. Antibodies activating immune stimulators, concurrent use of antibodies and cytokines, and administration of histidine-rich glycoprotein are currently applied solutions for macrophage polarization for antitumor effects while maintaining the total macrophage level [215,216,217]. However, further studies are required to identify specific molecules expressed in TAMCs within tumor tissues or peripheral blood in cancer patients.

In conclusion, TAMCs, including TAMs and MDSCs, play an important role in the progression, metastasis, and treatment responses in breast cancer via immune and/or nonimmune mechanisms. Effective therapeutic strategies targeting TAMs/MDSCs could be effective for breast cancer and as effective immune-based therapies.

## Figures and Tables

**Table 1:** Role of tumor-associated macrophages and myeloid-derived suppressor cells in breast cancer.

Cell Types	Roles	References
TAM-Immune mechanism	*Suppress CD8^+^T-cell activation by IL-10 *Repress effector T-cells by reduced L-arginine level by Arg1 *Inhibit T-cell response in murine mammary tumor by Arg1 and iNOS *Tumor immune invasion in mouse breast tumor by decreased IL-12 and iNOS *Decreased antigen presentation from reduced MHC class II expression	[61,62] [164] [65] [66,67] [68]
TAM-Nonimmune mechanism	*Promote angiogenesis by VEGF overexpression *Correlation between TAM infiltration and increased angiogenesis in human breast cancer *Secret increased amounts of angiogenesis mediator transcripts in the late stage mammary cancer *Promote breast cancer progression by ECM remodeling via uPAR expression in human breast cancer *Produce type I collagen in mouse breast tumor *Activate cancer stem cell by IL-6 *Enhance tumor sphere formation in co-culture of ER-positive breast cancer cell line and M2 macrophages *Interact with breast CSC and enhanced tumor formation and maintenance of CSC *Contribute mammary tumor intravasation by interaction between EGF from TAM and CSF1 from breast cancer cells *Promote breast cancer cell intravasation by integrin clustering via CCL18 *Involve lung metastasis of breast cancer by CD11b-positive macrophage recruitment by CCL2 *Enhance metastasis by CXCL1 in breast cancer cells	[74] [75,76] [25] [77] [78] [79] [80] [82] [86,87] [88] [92] [93]
TAM-Metabolic interaction	*Increase breast cancer cell proliferation by ARG1-mediated polyamine production *Increase breast cancer cell proliferation by increased secretion of lipocalin *Enhance breast cancer growth through heme oxygenase-1 in mouse model	[96] [165] [107]
TAM-Treatment resistance induction	*Increased treatment response of docetaxel in depletion of M2 TAM and expansion of M1 TAM in breast cancer *Induce treatment resistance in breast cancer though IL-10/STAT3/Bcl-2 signaling pathway in TAM [111] and secretion of chemoprotective molecules like cathepsin B and S in TAM *Result in resistance to antiangiogenesis agent by fibroblast growth factor, chemokine CCL18, thymidine phosphorylase, urokinase-type plasminogen activator (uPA), adrenomedullin (ADM), and semaphorin 4D (Sema4D) in TAM	[110] [113] [114]
MDSC-Immune mechanism	*Inhibit CD8^+^T-cells through granulocytic MDSC activated by IL-17 in breast cancer	[137]
MDSC-Nonimmune mechanism	*Inhibit PTEN and activate AKT pathway by activated miRNA-494 in MDSCs in breast cancer cell *Enhance breast cancer stemness through Notch and STAT3 activation by NO release *Promote breast cancer invasiveness and metastasis through STAT3 activation by IL-6 and IL6Rα and by increased AKT pathway and MMP expression *Induce EMT, tumor cell migration, and invasion through PI3K-AKT-mTOR pathway activation by MDSC in breast cancer cells *Promote osteolytic bone metastasis by acting as osteoclast progenitors *Promote metastasis through MMP and TGF-β1 by Gr-1^+^CD11b^+^ myeloid cell recruitment *Involve in extravasation, seeding, and tumor cell growth by differentiating monocytic MDSC into metastasis-associated macrophages *Promote EMT, tumor cell proliferation, and metastasis by secreting versican in CD11b^+^Ly6C^high^ monocytic MDSCs and by secreting TGF-β, VEGF, and IL-10 *Enhance cancer stemness in TNBC by MMP9 and chitinase 3-like 1 secretion	[52] [143] [46,144] [145] [49,146,147] [124] [148] [149,150] [151]

**Table 2:** Treatment target to tumor-associated myeloid cells for breast cancer.

Category	Agent	Subject	Mechanism	References
Treatment Target to Tumor-Associated Macrophages				
Inhibition of TAM Recruitment	carlumab (CNTO888)	Human clinical trial	Monoclonal antibody against CCL-2	[166,167]
	Anti-cathepsin D antibody	Mouse PDX model	Suppress TAM recruitment by TGFβ reduction	[168]
TAM killing	Trabectedin	Mouse tumor model	Caspase 8-dependent apoptosis via TRAIL receptors	[169]
	M2pep	Mouse TAM model	Pro-apoptotic peptide showing selective reduction of TAM	[170]
Modulator of TAM polarization	RG7155	Human breast cancer tissue	Monoclonal antibody to CSF1 tyrosine kinase receptor	[171]
	Zoledronic acid	Human clinical trial	Change M2 TAM into M1 TAM phenotype and inhibited carcinogenesis	[172]
	cGAMP-NP	Mouse xenograft model	Reprograming from protumorigenic M2-like phenotype toward M1-like phenotype	[173]
	Fbln7-C	Mouse model	Reprogramming of human monocytes into immunosuppressive TAMs	[174]
	Anti-CD40 mAbs	Mouse xenograft model	Induce M1 TAM polarization	[175]
	thymosin-α and β-glucan	Mouse model	Induce M1 TAM polarization	[176]
	YDW11	In vitro cell line	Inhibit M2 TAM polarization and cancer cell migration	[177]
Reduction of TAM products	XIAOPI formula	In vitro cell line and in vivo mouse xenograft	Inhibit CXCL1 from TAM and decrease premetastatic niche formation	[178]
	ZnPPIX	mouse xenograft model	Inhibit Heme oxygenase-1 from TAM	[107]
Treatment target to myeloid-derived suppressor cells				
Inhibition of MDSC formation and recruitment	Curcumin	TNBC model 4T1	Blocked IL-6 secretion and resulted in the reduction of number of MDSC	[179]
	BMP4	human and mouse breast tumor cell lines	TGF- β growth factor family, reduced G-CSF in breast cancer cell	[48]
	R84	In vivo mouse model	VEGF inhibitor to decrease the expression of IL-1β, IL-6 and CXCL1	[47]
	Sulforaphane and SB-265610	Mouse model 4T1	Inhibit MIF and CXCR2, and suppress MDSC formation and migration	[51,53]
	Silbinin	mouse xenograft model	Inhibit CCR2 expression in MDSC and block MDSC recruitment in tumor site	[180]
	L-NMMA	In vitro cell line	Inhibit MDSC-mediated osteolysis by blocking the differentiation of MDSC into osteoclast	[49]
	HuMax-IL8	In vitro cell line and in vivo mouse xenograft	Reduce granulocytic MDSC recruitment, and enhance NK and T cells immune-mediated killing	[181]
Elimination of MDSC	aATC	In vitro cell line	Deplete MDSC in breast cancer	[182]
	Listeria^at^	Mouse model 4T1	Infect MDSC and reduced the number of MDSC	[183]
	herpes simplex virus 1 vector with 15-PGDH	breast cancer mouse model	Reduction of number of MDSC through conversion of prostaglandin E2 into inactive 15-keto-metabolites	[184]
	STING ligand	Mouse model 4T1	Phenotype change of MDSC to repress the function of MDSC	[185]
	Doxorubicin	murine mammary cancer model	Eliminate MDSC by MDSC apoptosis through ROS system	[186]
	celecoxib	in vivo mouse xenograft	COX-2 inhibitor to decrease the number of MDSC	[187]
Reduction of MDSC products	1-MT	In vitro breast cancer cell line	Repress IDO from MDSC and result in repression of immune suppressive function of MDSC against T-cell	[188]
	NOV-22	Human clinical trial	Glutathione disulfide mimetic to inhibit ROS and reverse MDSC role to repress CD8+T cell response	[130]

TAM, tumor-associated macrophage; CCL, chemokine (C-C motif) ligand; TRAIL, TNF-related apoptosis-inducing ligand; CSF, Colony-stimulating factor; TLR, Toll-like receptors; CXCL, C-X-C motif chemokine; MDSC, myeloid-derived suppressor cell; BMP, Bone morphogenetic protein; MIF, Macrophage migration inhibitory factor; CXCR, CXC chemokine receptors; CCR, CC chemokine receptors; L-NMMA, L-nitromonomethylarginine; 15-PGDH, 15-hydroxyprostaglandin dehydrogenase; IDO, Indoleamine 2,3-dioxygenase; 1-MT, 1-methyl-L-tryptophan.

## Abbreviations

ADM	adrenomedullin
ALDH	aldehyde dehydrogenase
BMP	bone morphogenetic protein
bsAb	bispecific antibody
CCL	C-C chemokine ligand
CCR	C-C chemokine receptor
c-di-GMP	cyclic di-guanylate
COX	cyclooxygenase
CSC	cancer stem cell
CTLA-4	cytotoxic T-lymphocyte-associated protein 4
CXCL	chemokine (C-X-C motif) ligand
DCIS	ductal carcinoma in situ
ECM	extracellular matrix
E-FABP	epidermal fatty acid-binding proteins
EGF	epidermal growth factor
EGFR	epidermal growth factor receptor
EMT	epithelial-mesenchymal transition
EphA4	EPH receptor A4
GAMP	guanosine monophosphate–adenosine monophosphate
GM-CSF	granulocyte-macrophage colony-stimulating factor
HER2	human epidermal growth factor receptor 2
HIF	hypoxia-inducible factor
HO-1	heme oxygenase-1
HSP	heat shot protein
IDC	invasive ductal carcinoma
IDO	indoleamine 2,3-dioxygenase
IFN	interferon
IL	interleukin
iNOS	inducible nitric oxide synthase
LAG-3	lymphocyte activation gene-3
LCN	lipocalin
L-NMMA	NG-monomethyl-L-arginine acetate
LOX	lysyl oxidase
LPS	lipopolysaccharide
MDSC	myeloid-derived suppressor cell
MHC	major histocompatibility complex
MIF	macrophage migration inhibitory factor
MMP	matrix metallopeptidase
MMR	macrophages mannose receptor
MPO	myeloperoxidase
MRC1	mannose receptor C-type 1
mTOR	mammalian target of rapamycin
NF	nuclear factor
NK	natural killer
NO	nitric oxide
OS	overall survival
PD-L1	programmed death-ligand 1
PI3K	phosphoinositide 3-kinase
PTEN	phosphatase and tensin homolog
RFS	relapse-free survival
RNI	reactive nitrogen intermediates
ROS	reactive oxygen species
SDF-1	stromal cell-derived factor 1
Sema4D	semaphorin 4D
SOCS1	suppressor of cytokine signaling 1
SOX2	SRY-box transcription factor 2
STAT	signal transducer and activator of transcription
STING	stimulator of interferon genes
TADC	tumor-associated dendritic cell
TAM	tumor-associated macrophages
TAMC	tumor-associated myeloid cell
TAN	tumor associated neutrophil
TCR	T-cell receptor
TEM	Tie2-expressing monocytes
TGF	transforming growth factor
TGM2	transglutaminase 2
TLR	toll-like receptor
TME	tumor microenvironment
TNBC	triple-negative breast cancer
TRAIL	TNF-related apoptosis-inducing ligand
uPA	urokinase-type plasminogen activator
uPAR	urokinase receptor
VEGF	vascular endothelial growth factor
VEGR	vascular endothelial growth factor receptor
1-MT	1-methyl-L-tryptophan
15-PGDH	15-prostaglandin dehydrogenase
